# Learn to fly: Training and competencies to support the multidisciplinary workforce needs of learning health systems

**DOI:** 10.1002/lrh2.10347

**Published:** 2022-10-02

**Authors:** Sarah M. Greene, Kristi L. Holmes

**Affiliations:** ^1^ NAM Leadership Consortium National Academy of Medicine Washington DC USA; ^2^ Department of Preventive Medicine, Galter Health Sciences Library and Learing Center Northwestern University Feinberg School of Medicine Chicago Illinois USA

The concept of the learning health system (LHS) originated in the mid‐2000s through a series of workshops and publications^1^, ^2^, ^3^ produced by the National Academy of Medicine (NAM, formerly the Institute of Medicine). Spurred by the urgency to generate and mobilize research evidence to improve health and healthcare, thought leadership from the NAM has galvanized the field. In just 15 years, the LHS concept has spurred the development of a robust bibliography of ways to address important gaps and deficits, spawned a dedicated journal, and infused new thinking about how to harness electronic health data to support continuous learning. Most importantly, the LHS has catalyzed remarkable opportunities for community and capacity building by providing opportunities to nurture a new generation of multidisciplinary LHS practitioners in this exciting and evolving field.

The field has enjoyed remarkable growth and maturation over the past several years, as “internal data and experience (are increasingly) and systematically integrated with external evidence, and that knowledge is put into practice.”^4^ The LHS workforce is critical to its success and underscores the need for workforce training and related competency building approaches to support the development and sustainability. This Special Issue offers the LHS community an opportunity to focus on our workforce—and their broad professional roles, skills, training, expertise, and lived experience—as we identify and consider academic training and professional skills needed to successfully close the gap from discovery to the use of knowledge in practice.

This work is happening at all levels, from the individual health system and academic institution level to national and international initiatives. The National Institutes of Health (NIH) has funded dedicated “collaboratories” that engage health systems and academic researchers to develop pragmatic research with high potential for implementation and adoption at scale. The Agency for Healthcare Research and Quality (AHRQ) and the Patient‐Centered Outcomes Research Institute (PCORI) have established a training grant program with the purpose of “train[ing] clinical and research scientists to have the skills to support and lead efforts to apply patient‐centered outcomes research (PCOR) methods and conduct PCOR research in a LHS and to facilitate rapid implementation of evidence that will improve quality of care and patient outcomes.”^5^ These changes are also being catalyzed at the local level. The dedicated department of Learning Health Sciences at the University of Michigan is both a harbinger and a blueprint for other academic institutions. Curricula at newer schools of medicine, including the Geisinger Commonwealth School of Medicine and Kaiser Permanente's Bernard J. Tyson School of Medicine, endorse and espouse the importance of the LHS. In other institutions, training opportunities may live in departments of biomedical informatics, health services research, or other parts of the organization—again underscoring the requisite multidisciplinary and complementary capabilities that are needed to study and improve the delivery of healthcare. Taken together, these efforts to fund and instantiate new scholarly endeavors are key signals that building the workforce is critical to realizing the full potential of the LHS. This Special Issue features a range of training programs and perspectives representing different countries, systems, organizational structures, and topical areas to inform and inspire.

Three articles in this Special Issue of the *Journal* examine competency development and extension in support of LHS training. In “Competency Analysis and Educational Strategies to Meet the Demand for a Learning Health System Workforce,” Feldman et al.^6^ offer direction for the development of LHS programs, including approaches to identify and address challenges during program development, and strategies to guide both new program development and collaboration across existing programs. Such direction is critical for continued field‐building efforts, insofar as newer training programs will not need to start from a blank slate. Coley and colleagues,^7^ through the Consortium for Applied Training to Advance the Learning health system with Scholars/Trainees (CATALyST) K12 program, further advance this discussion in their commentary, “A Call to Integrate Health Equity into Learning Health System Research Training.” The authors advocate persuasively for competency domains to be extended to reflect health equity‐focused LHS science. Through real‐life case studies, program components, challenges, and potential solutions are illustrated at the program, funder, and research community level. Training and mentorship in health equity‐focused LHS science underscore the importance for the LHS field to recognize and build upon existing health equity work, particularly by scholars of color. Notably, the LHS K12 funders and program leaders readily adopted Equity and Justice as an eighth core domain of the LHS K12 training program.

While both the Feldman and Coley commentaries offer guidance for the development of resources for such programs. Franklin and colleagues^8^ describe novel efforts to instill a consistent evaluation approach for scholars' relative mastery of the LHS domains in their Experience Report, “Development of a Learning Health System Science Competency Assessment to Guide Training and Proficiency Assessment.” Here, national experts were interviewed to better characterize proficiency in competencies across the seven domain areas that comprise the pillars of the AHRQ‐PCORI LHS K12 program (ie, systems science; research questions and scientific evidence; research methods; informatics; ethics of research and implementation; improvement and implementation science; and engagement, leadership, and research management), resulting in an LHS Competency Assessment. The assessment supports the LHS workforce in prioritizing and monitoring proficiency development for individuals and within training programs. It is both important and prescient for the burgeoning LHS field to have approaches to gauging expertise, given the complexity of the LHS and the skills needed for effective execution.

Workforce development programs play a critical role in the LHS ecosystem. Dushyanthen et al.^9^ report on an innovative “LHS Academy” fellowship program for interprofessional clinicians to support workforce needs in “Fostering the Use of Learning Health Systems through a Fellowship Program for Interprofessional Clinicians.” This fellowship program lasts less than a year and consists of coursework and projects to nurture participants' roles as “digital health champions.” Program evaluation captured feedback about the program and also probed participants' reflections on their perceived barriers to participation, application of learned content in the workplace and in their work, and recommendations for program improvement. In “Review of Applied Health Informatics Courses in a Multidisciplinary Biomedical Informatics Department,” Motiwala et al.^10^ focus on the critical need for workforce development efforts to support informatics infrastructure in LHS. Like Dushyanthen and colleagues, they leverage survey‐based program evaluation to assess their interdisciplinary applied health informatics program to assess the program and faculty to develop strategies for program expansion.

The Experience Reports included in this Special Issue offer a bird's‐eye view of programs and partnerships positioned across a range of organizations and communities of practice focused on planning, implementing, operating, or improving workforce development for LHS. Notable efforts by AHRQ and the US Veterans Affairs (VA) Health System feature prominently. In addition to the aforementioned work by Franklin and colleagues, Lozano et al.^11^ summarize the approaches, challenges, and successes experienced individually and collectively by the K12 training programs that were awarded through the AHRQ‐PCORI national centers of excellence program alluded to above. This distributed and collaborative approach fostered a range of partnerships for these centers and scholars, including not just health systems, but also VA health centers and safety net providers. Program scholars hailed from both clinical and non‐clinical training backgrounds and were given the opportunity to expand knowledge into practice through outreach and research experiences.

The VA is an important and noteworthy example of a mature LHS in action. In this issue, the VA's extensive LHS training ecosystem, as described by Kilbourne and colleagues in “How the VA is Training the Next‐Generation Workforce for Learning Health Systems,” explores workforce requirements in the context of research and clinical goals, funding, infrastructure, and collaboration.^12^ Rural clinical workforce capacity building through an adapted training program is described in “Implementation Experience and Initial Assessment of a Rural Women's Health Training Program in Support of the U.S. Department of Veterans Affairs (VA) as a Learning Health System.”^13^ Here, Sanders and colleagues describe the implementation and effectiveness of a women's health rural workforce training program delivered at rural sites, sharing lessons learned that can be considered for other training programs. This work is an important contribution to the discussion about capacity building beyond the urban academic medical environment and shows how LHS programs can also be tailored to support trainees with specialized backgrounds and/or clinical interests.

The VA also offers the full LHS workforce to learn and benefit from quality improvement (QI) initiatives. In “Continuous Quality Improvement at the Frontline: One Interdisciplinary Clinical Team's 4‐year Journey After Completing a Virtual Learning Program,” Robinson and colleagues^14^ describe their experience and outcomes from a virtual coaching program and hands‐on curriculum designed to support capacity building for continuous QI activities, a hallmark of LHSs. A unique partnership to support QI is described in the article by Vilendrer and colleagues^15^: “Evaluating Clinician‐Led Quality Improvement Initiatives: A System‐Wide Embedded Research Partnership at Stanford Medicine.” This article describes engagement and partnership between the unit charged with coordinating clinician‐led QI projects with an in‐house implementation and evaluation unit. Finally, Masica and colleagues^16^ offer readers an opportunity to consider LHS workforce development in the context of translational science in “The Texas Health Resources Clinical Scholars Program: Learning Health Care System Workforce Development through Embedded Translational Research.” Here, a collaboration between a large, non‐profit health system and a large academic medical center to launch a Clinical Scholars Program supports workforce development focused upon later‐stage translational research and engagement with community partners. The report highlights program design and operational components, which serve as guidance for others wishing to build upon similar partnerships to support LHS workforce development.

Taken as a whole, this Special Issue offers an excellent starting point to understand the current state (and desired future state) for LHS workforce development across a range of initiatives in academic and real‐world clinical settings. Given its relatively recent emergence as a field, the LHS is undergoing rapid yet thoughtful maturation, including training and development of the LHS workforce. Even so, there is still much yet to learn and explore, including

- How do we define success at the level of the trainee, the trainers, institutions, and the healthcare system writ large?
- How can we ensure that the LHS and its workforce mature into exceptional leaders who can drive meaningful and much‐needed change to ensure a system of continuous learning that works for all?
- Can training programs be readily adapted and reused or do we need de novo work for each institution, system, or setting?
- How can we ensure that trainees are adequately versed in working with system leaders, as well as patients, communities, and other stakeholders?
- How can we identify and address persistent and emerging gaps in competencies such as communication and engagement, impacts of the business of healthcare, and health equity and social justice issues across our communities?

Efforts to optimize the LHS workforce must also be placed in the context of the overall healthcare workforce, which itself is addressing tectonic changes, including workforce shortages and moral injury, and substantive changes to the organization, delivery, and financing of healthcare. Evidence generation and application are central to these efforts but may be difficult to embed in a healthcare climate that is occasionally or consistently ambivalent toward research.^17^ Reconsideration of roles, scope of practice, and licensure are likely to occur sooner rather than later as these questions—and changes—are considered and addressed.

As this Special Issue illustrates, the importance of collaboration throughout the learning and competency development process cannot be overstated. In the Foo Fighters' 1999 hit rock song, “Learn to Fly,” Dave Grohl sings, “Fly along with me, I can't quite make it alone.” That collaborative spirit permeates this body of contributed works and the entire LHS ecosystem. For all learning health system efforts to reach their full potential, we need to fly together and assure that the workforce has the full breadth of capabilities to fulfill the virtuous cycle of using evidence at scale for transformative improvements to health and healthcare.
